# Savant syndrome has a distinct psychological profile in autism

**DOI:** 10.1186/s13229-018-0237-1

**Published:** 2018-10-12

**Authors:** James E A Hughes, Jamie Ward, Elin Gruffydd, Simon Baron-Cohen, Paula Smith, Carrie Allison, Julia Simner

**Affiliations:** 1 School of Psychology, Pevensey Building, University of Sussex, Brighton, BN1 9QJ UK; 2 Autism Research Centre, University of Cambridge, Cambridge, CB2 8AH UK

**Keywords:** Autism spectrum conditions, Savant syndrome, Sensory processing, Cognition, Perception, Talent, Skill learning

## Abstract

**Background:**

Savant syndrome is a condition where prodigious talent can co-occur with developmental conditions such as autism spectrum conditions (autism). It is not yet clear why some autistic people develop savant skills while others do not.

**Methods:**

We tested three groups of adults: autistic individuals who have savant skills, autistic individuals without savant skills, and typical controls without autism or savant syndrome. In experiment 1, we investigated the cognitive and behavioural profiles of these three groups by asking participants to complete a battery of self-report measures of sensory sensitivity, obsessional behaviours, cognitive styles, and broader autism-related traits including social communication and systemising. In experiment 2, we investigated how our three groups learned a novel savant skill—calendar calculation.

**Results:**

Heightened sensory sensitivity, obsessional behaviours, technical/spatial abilities, and systemising were all key aspects in defining the savant profile distinct from autism alone, along with a different approach to task learning.

**Conclusions:**

These results reveal a unique cognitive and behavioural profile in autistic adults with savant syndrome that is distinct from autistic adults without a savant skill.

**Electronic supplementary material:**

The online version of this article (10.1186/s13229-018-0237-1) contains supplementary material, which is available to authorized users.

## Background

People with savant syndrome are characterised by their remarkable talent in one or more domains (e.g. music, memory) but also by the presence of some form of developmental condition such as autism spectrum conditions (henceforth autism) [1]. Autism describes a set of symptoms involving difficulties in social communication, unusually repetitive/routine behaviours, unusually narrow interests, and atypical sensitivity to sensory stimuli [2]. Recent models of autism also focus on strengths associated with the condition (not just on their difficulties), in areas such as perceptual and cognitive processing [3], systemising [4], and attention to detail [5], as well as areas of interest, aptitude, and talents. In savant syndrome, talents and skills observed in such individuals far exceed their own overall level of intellectual or developmental functioning.

Exceptional cases of *prodigious* savant syndrome occur when an autistic individual’s level of skill goes beyond that seen even in the general population. A well-known example of a prodigious savant is the artist Stephen Wiltshire who is capable of drawing hyper-detailed cityscapes from memory and who also has autism [6]. Savant skills can exist in a variety of areas, but most savants show skills in art (e.g. hyper-detailed drawings), music (proficiency in musical instrument playing), maths (fast mental arithmetic), calendar calculation (the ability to provide the day of the week for any given date), and memory recall of facts, events, numbers etc. [7].

Although savant syndrome can co-occur with a range of developmental conditions, most cases involve autism in some form [8, 9] and savant syndrome has been reported to occur in up to 37% of autistic individuals [10]. The emergence of savant skills in autistic adults is not fully understood, and there is a lack of empirical evidence to support current theories. The motivation for the current research is to understand the condition of savant syndrome in more depth by contrasting a group of autistic savant individuals with a group of autistic individuals who do not have a savant skill. A third group of typical controls without autism or savant skills serve as a comparison. With this approach, we aim to separate features that are tied to savant syndrome from features that are tied to autism per se. We ask what individual differences lie within the autistic population that might allow some to develop savant skills while others do not. We first summarise current theoretical frameworks on the origins of savant skills. We then present two experiments that consider the development of savant skills at multiple levels of cognition, perception, and behaviour.

There is no consensus on exactly how savant skills are developed in autistic individuals. Bölte and Poustka [11] showed that savants do not show differences in standard intelligence compared to other autistic individuals. It could therefore be that their skills develop simply through many hours of extended practice. This would be similar to the abilities of neurotypical ‘memory athletes’ who can, for instance, memorise thousands of digits of pi using mnemonic techniques, with top performers relying on thousands of hours of practice—as in other sports [12–14]. Savants too appear to require practice, but here we ask exactly *why* they practice and whether they also have cognitive or perceptual differences beyond practice alone.

Two theoretical models have bridged the gap between need-for-practice and autistic symptoms in savants [15, 16]. Happé and Vital [15] proposed that one way in which savant skills might emerge could be through the autism-related trait of mind-blindness, which is the difficulty in attributing mental states to others [17, 18]. Happé and Vital [15] suggest that a lack of interest in the social world could serve to free up cognitive and time resources that are usually dedicated to monitoring social interactions. As a result, these extra resources could be re-allocated to the development of talent by permitting more time (i.e. practice) to the nurturing of restricted interests commonly observed in autistic individuals. Since these cognitive resources have been allocated away from monitoring social interactions, a further expected consequence might also be lower social and communication skills in savants and we explore this in experiment 1 below.

In contrast, Simner et al. [16] suggest that the hours spent achieving savant ability are the result not of mind-blindness, but of the autism-linked trait of obsessiveness—i.e. savants have an obsessive urge to over-rehearse their skills to prodigious levels. Tentative support for this comes from LePort et al. [19] who showed that a group of individuals with prodigious event-memory (some of whom are likely to be savants [16]) showed higher obsessional traits than controls. However, the controls they tested did not have autism, making it unclear whether obsession was tied to savant skills per se or simply to autism (or other co-occurring neurodevelopmental differences [20]). O’Connor and Hermelin [21] compared savants to controls with autism and drew similar conclusions about obsessiveness—but their questionnaire also contained items unrelated to obsessions (e.g. decision-making). In addition, they may not have corrected their question-by-question statistics for multiple comparisons, making it difficult to tie their findings to any particular trait. Similarly, Howlin et al. [10] used a questionnaire of just five questions, testing repetitive behaviours with a number of other traits (e.g. sensory sensitivity), again making it difficult to interpret their findings (of no difference between autistic-savants and autistic-nonsavants).

Finally, Bennet and Heaton [22] found higher scores for savant children on a five-question factor they named ‘obsessions and special interests’ compared to autistic-nonsavants, but traced this back to an individual question related to becoming absorbed in different topics. Given these differences across studies in their focus, questionnaire length, and testing groups, it remains unclear whether savants are particularly notable for their obsessional traits, above and beyond what we would expect from autism alone. Here we test both models described above, i.e. to see whether savants are particularly notable for their obsessional traits or for traits that are linked to mind-blindness (e.g. social and communication skills), compared to autistic individuals without savant skills.

Although both types of rehearsal (from mind-blindness or obsessiveness) could influence savant skills, this practice alone probably does not act as the only catalyst for talent to emerge. There may also be differences in certain cognitive abilities, linked to autism, which manifest themselves more strongly in individuals who acquire savant skills compared to those who do not. Specifically, we propose here and previously [16, 23] that talent could emerge from autism traits such as excellent attention-to-detail, hyper-systemising, and sensory differences. For example, the combination of attention-to-detail and hyper-systemising may predispose some autistic individuals to develop talent through the increased detection of ‘if p, then q’ rules [23]. These rules can be found in savant skills such as calendar calculation (i.e. stating the weekday for a given date) and can be learned from predictable patterns within the calendar itself.

A related proposal is Mottron et al.’s [24] ‘veridical mapping’ that links savant talent to the enhanced ability of autistic individuals to detect regularities within and between systems. Some savant skills do indeed depend on mapping regularities across systems (e.g. mapping from musical pitch to note-label in absolute pitch). In addition, savants appear to show a particular cognitive style of enhanced local processing, as outlined in the *enhanced perceptual functioning* model [3], and less global interference (e.g. in a target-detection task [25]) at least when activities demand active interaction [26]. Again, however, it is not clear whether these influences are tied to being a savant or simply having autism. Here we test groups of autistic individuals with and without savant syndrome to examine whether savants have a particular cognitive style (e.g. local bias), as well as elevated autism-related traits such as systemising.

Savant talent may also have important sensory components. Baron-Cohen et al. [23] argue that heightened sensory sensitivity may be the pre-requisite for excellent attention-to-detail, which they theorise as an autistic trait linked to savant syndrome. Subjective accounts of sensory irregularities in autism have been shown previously [27–30], and multiple studies have objectively demonstrated superior visual, auditory, and tactile sensory perception in autism [31–36]. These sensory differences may bring about the emergence of talent by affecting information processing at an early stage [23] although this suggestion is not universally supported [22].

One final sensory link between autism and savant syndrome is the presence of synaesthesia, where stimuli such as letters, numbers, and sounds invoke automatic and additional sensory experiences such as colours [37, 38]. Hughes et al. [39] found that synaesthesia occurs at higher levels among autistic individuals with savant skills (but not those without savant skills). Simner et al. [37] hypothesised that the obsessive over-rehearsal of savants may focus particularly on skills born out of synaesthesia, building on earlier work [25]. Elsewhere, we have already supported one branch of this model by showing that people with synaesthesia have elevated skills in savant domains (e.g. event recall [16]). Here we test the other branch of the model by examining whether their rehearsal is born out of obsessive traits [16] or mind-blindness which might predict lower social or communication skills [15]. Finally, we test the role of sensory sensitivities more generally, by comparing the sensitivities of autistic individuals with and without savant skills.

In our experiments, we look at two groups of autistic individuals, with and without a savant skill (specifically, *prodigious* talents which are above the skills of the general population). In experiment 1, we contrast our groups on cognitive and sensory self-report measures predicted by previous theoretical accounts. We test differences related to sensory sensitivity using the Glasgow Sensory Questionnaire (GSQ) [30], we test obsessive-behaviours using the Leyton Obsessional Inventory (LOI) [40], we test cognitive styles (e.g. local bias) using the Sussex Cognitive Styles Questionnaire (SCSQ) [41], and we test autistic traits such as systemising using the Systemising Quotient-Revised (SQ-Revised) [42] and the Autism Spectrum Quotient (AQ) [43]. In addition to our two groups of autistic individuals, with and without savant skills, we also test a typical control group with neither autism nor prodigious talents.

As stated above, there is very little empirical evidence to evaluate current theories of savant syndrome apart from tentative pointers towards increased obsessionality [16] and evidence for links to synaesthesia [16, 39]. Our goal is to test all theories directly; therefore, our predictions are based on the above theoretical frameworks. Following the theory by Baron-Cohen et al. [23], we predict that savants, relative to autistic individuals without a savant skill, will report more traits or behaviours related to sensory sensitivity, attention-to-detail, and systemising. We also predict they will report a more local (as opposed to global) cognitive style since this has previously been implicated in (e.g. visual search) advantages in autism and has been theorised to contribute to the development of savant skills [44]. Based on the model of autism-linked obsessive rehearsal [16], we predict that autistic-savants will report more obsessional behaviours compared to autism individuals without a savant skill. Alternatively, the rehearsal account based on mind-blindness [15] predicts that autistic savants would have lower social or communication skills (here measured using the AQ) compared to autistic individuals without a savant skill. Finally, we predict that both autism groups, regardless of the presence of a savant skill, will report heightened traits or behaviours in all of the above areas compared to the typical control group.

Experiment 2 investigates how a distinct psychological or behavioural profile in savants (explored in experiment 1) might influence performance on a behavioural task. We test the same three groups, to determine whether savants have a particular style of learning when presented with a novel savant skill: calendar calculation*.* As noted above, calendar calculation is the ability to give the correct day of the week for a given date in the past or future (e.g. 18^th^ September 1990 was a Tuesday) and is considered one of the most characteristic savant abilities [7]. In experiment 2, three groups of participants (autistic-savants, autistic-nonsavants, controls) learned how to calendar calculate through a series of tutorials about different patterns and rules of the calendar. It is unclear whether calendar-calculating savants rely on rote memorisation of dates [45] or internalisation of the inherent rules of the calendar (e.g. 1^st^ March 2013, 2014, 2015 = Friday, Saturday, Sunday respectively) or indeed whether they use some multi-faceted approach [44]. No studies to date have investigated the learning of calendar calculation skills in savants (who do not already possess this skill) compared to nonsavant autistic individuals and controls; therefore, our predictions below are again based on current theoretical models of savant syndrome.

If savant syndrome is linked to pre-existing abilities or dispositions (as opposed to practice alone), then we predict that savants may show a superior level of accuracy. In particular, the ‘enhanced perceptual functioning’ and ‘veridical mapping’ models predict more accurate performance by savants from their superiority in learning pattern/rule-based skills [3, 24, 44]. In contrast, accounts of savant skills that emphasise obsession or practice may not predict immediate advantages without extended training but might predict a different learning approach. Thus, if savants show increased repetitive/obsessive tendencies, we might expect them to engage in a slower, more careful approach to our calendar calculation task from, for example, increased answer checking.

In summary, our studies investigate savant syndrome by directly contrasting savants against a group of autistic individuals without a savant skill as well as a typical control group. Our investigation is the first to take an empirical approach to test a number of theoretical accounts of savant syndrome [15, 16, 23, 24, 44], some of which currently lack a clear empirical foundation.

## Experiment 1: traits linked to savant syndrome

### Methods

#### Participants

One hundred and eleven participants took part in the study. They comprised 44 autistic individuals with savant skills (‘autistic-savants’: 23 female; mean age 36.52, range 20–55, SD = 9.56), 36 autistic individuals without a savant skill (‘autistic-nonsavants’: 23 female; mean age 36.67, range 18–51, SD = 9.35), and 31 typical controls with neither autism nor a savant skill (‘controls’: 25 female; mean age 36.84, range 18–50, SD = 10.94). Participants were matched group-wise on age, with no significant differences across groups *F*(2, 110) = .009, *p =* .991.

Participants were recruited from two sources. Three of the 44 autistic-savants were recruited from The Savant Network, which is a group of individuals with a self-reported savant skill who have expressed an interest in taking part in research studies at the University of Sussex. The remaining autistic-savants were recruited from the Cambridge Autism Research Database (CARD). All autistic-nonsavant individuals and all controls also came from CARD, which holds status information of both autism and typical participants. To ensure that our autism participants had sufficient cognitive levels to independently provide consent, we sent our recruitment materials to high functioning autistic adults, as detailed in the CARD database of autistic participants. Participants volunteered to take part in our study in response to an email advertisement that was sent to 4172 participants in these databases (553 autistic-savants, 930 autistic-nonsavants, and 2689 typical adults). The email did not describe the nature of our tests but invited participants to take part in studies that look into how people ‘perceive and interact with the world around them’. Participants did not receive payment for taking part, and our study was approved through the Cross-Schools Science and Technology Research Ethics Committee at the University of Sussex. In addition to the 111 participants, we additionally recruited but subsequently excluded 12 further participants because they initially indicated autism but failed to meet our criteria when probed further (see the ‘Procedure’ section).

All individuals in the autism groups (autistic-savant; autistic-nonsavant) self-reported having a formal diagnosis of autism in our questionnaire (see the ‘Materials’ section): 9 autism, 64 Asperger syndrome, 1 pervasive developmental disorder not otherwise specified, and 6 other. These formal diagnoses had also been recorded for 77 of the 80 autistic individuals as part of their CARD recruitment procedure. There were no controls who reported autism. All autistic-savants, and no other group, self-reported having a savant skill (in our *Sussex Savant Questionnaire*; see below).

### Materials

We administered the following questionnaires: the Sussex Savant Questionnaire (SSQ), the Glasgow Sensory Questionnaire (GSQ) [30], the Leyton Obsessional Inventory—short form (LOI) [40], the Sussex Cognitive Styles Questionnaire (SCSQ) [41], the Systemising Quotient-Revised (SQ-R) [42], and the Autism Spectrum Quotient (AQ) [43]. These are described below.

### Sussex Savant Questionnaire

**Fig. 1:**
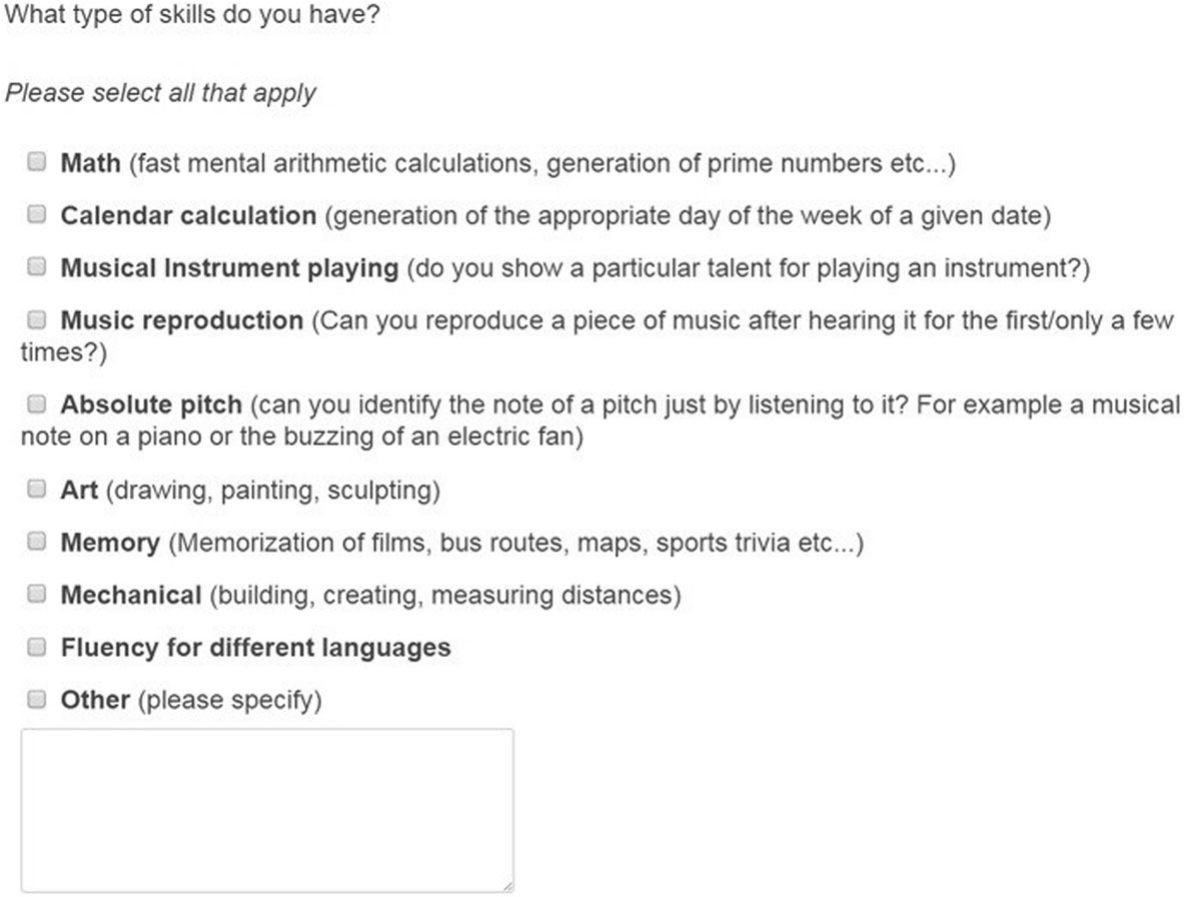
Savant skill categories, as presented during the savant skills questionnaire

This questionnaire was created for the purposes of this study. An initial question asked ‘Have you received a formal diagnosis of any of the following: Autism, Asperger Syndrome, Pervasive developmental disorder not otherwise specified; ‘other’?’. Next, we provided a definition of *prodigious* savant syndrome and then asked: ‘Do you think that you have any skills, abilities, or talents (e.g. art, maths, music etc.) that are beyond the abilities of the general population?’ Participants who responded in the affirmative to this question were given a list of nine categories of savant skills to choose from and used check boxes to indicate the skills that were relevant to them (see Fig. 1). One option was ‘other’ with a text-box provided for elaboration.

### Autism Spectrum Quotient

The AQ contains 50 items to measure autistic traits in adults of average or above average intelligence [43]. The AQ contains 10 statements for each of five different subscales: social skills, attention switching, attention-to-detail, imagination, and communication. Participants responded to each statement on a four-point scale (definitely agree, slightly agree, slightly disagree, definitely disagree). Example items included ‘I find it hard to make new friends’, ‘It does not upset me if my daily routine is disturbed’, and ‘I find it difficult to imagine what it would be like to be someone else’. Approximately half of the questions are reverse coded. Responses were coded as 0 or 1, with total scores ranging from 0 to 50. Items were given a score of one point if the participant recorded an autistic trait (e.g. exceptional attention-to-detail or poor social skill) using the ‘slightly’ or ‘definitely’ response. A total score of 32 or above is used is a strong indicator of likely autism [43].

### Systemising Quotient-Revised

The SQ-R contains 75 items with possible scores ranging from 0 to 150, where a higher score suggests a greater tendency to systemise. Systemising is defined as the drive to identify and analyse systematic relationships or patterns in rule-based information. Participants demonstrated their level of agreement with each statement using a four-point scale (definitely agree, slightly agree, slightly disagree, definitely disagree). An individual scores two points if he/she strongly displays a systemising response and one point if they slightly display a systemising response, and approximately half the items are reverse-coded. Example items included ‘When I look at a building, I am curious about the precise way it was constructed’ and ‘If I were buying a stereo, I would want to know about its precise technical features’.

### Glasgow Sensory Questionnaire

The GSQ contains 42 items (scored from 0 to 4, ‘never’ to ‘always’ respectively, with possible total scores ranging from 0 to 168) that explore unusual sensory behaviours, for example, ‘Do you react very strongly when you hear an unexpected sound?’ and ‘Do bright lights ever hurt your eyes or cause a headache?’. The questionnaire measures sensory sensitivity across seven modalities that include visual, olfactory, auditory, gustatory, tactile, vestibular, and proprioception. Each of these modalities is represented by six items in the questionnaire, and this is further broken down into three items each in order to measure both hypo-sensitivity and hyper-sensitivity per modality.

### Sussex Cognitive Styles Questionnaire

The SCSQ consists of 60 questions that assess the general cognitive profile of an individual and his/her style of thinking (e.g. visual/verbal cognitive styles). Each question has one of five answers (strongly disagree, disagree, neither agree nor disagree, agree, strongly agree). Each question is linked to one or more of six factors (imagery ability, technical/spatial abilities, language and word forms, need for organisation, global bias, and systemising). The factor of ‘imagery ability’ refers to the use of visual imagery in everyday life (e.g. ‘I often use mental images or pictures to help me remember things’). The factor ‘technical/spatial abilities’ refers to technical interests (e.g. ‘If I were buying a computer, I would want to know exact details about its hard drive capacity and processor speed’), mathematical abilities (e.g. ‘I am fascinated by numbers’), and the use of spatial mental imagery (e.g. ‘I can easily imagine and mentally rotate three-dimensional geometric figures’). The factor ‘language and word forms’ refers to an interest in the visual appearance of written language as opposed to spoken language abilities (e.g. ‘When I hear a new word, I am curious to know how it is spelled’; ‘When I read something, I always notice whether it is grammatically correct’). The factor ‘need for organisation’ refers to things relating to order and organisation (e.g. ‘If I had a collection (e.g. CDs, coins, stamps), it would be highly organised’). The factor ‘global bias’ refers to the tendency to process stimuli holistically rather than by its local features (e.g. ‘I usually concentrate on the whole picture, rather than the small details’). Reverse scored questions for this factor indicate more attention-to-detail or a local processing preference (e.g. ‘I tend to focus on details in a scene rather than the whole picture’). Finally, the factor ‘systemising tendency’ refers to an interest in systems (e.g. ‘I am fascinated by dates’) and categorisation (e.g. ‘When I look at an animal, I like to know the precise species it belongs to’).

### Leyton Obsessional Inventory—short form

The LOI consists of 30 questions that assess the presence or absence of obsessional symptoms using a ‘true/false’ format. Each question relates to one of four factors (contamination, doubts/repeating, checking/detail, and worries/just right) in the questionnaire. Factor 1—‘contamination’ is related to concerns about germs, dirty environments, obsessive cleanliness, and the excessive use of cleaning products (e.g. ‘I avoid using the public telephone because of possible contamination’). Factor 2—‘doubts/repeating’ is related to uncomfortable thoughts, repeating behaviours, checking, and serious doubts about everyday things (e.g. ‘I frequently get nasty thoughts and have difficulty getting rid of them’). Factor 3—‘checking/detail’ is specifically related to repeated checking, too much attention-to-detail, conscience/honesty concerns, and strict routine (e.g. ‘I am more concerned than most people about honesty’). Factor 4—‘worries/just right’ is related to behaviours such as taking a long time to dress and to hang up and put away clothing, worrying about bumping into other people, and belief in unlucky numbers (e.g. ‘some numbers are extremely unlucky’).

### Procedure

All participants were tested remotely via the online survey-hosting platform Qualtrics (www.qualtrics.com). Participants (autistic-savants, autistic-nonsavants, and controls) accessed the study by clicking on a URL provided to them electronically. After seeing the information sheet and consent page, participants saw the following questionnaires in order: SSQ, AQ, SQ-R, GSQ, SCSQ, and LOI. For those participants recruited from the CARD database, the AQ and SQ-R data were collected in a separate procedure as part of the standard protocol for participants when signing up to that database. In this, participants completed the AQ and SQ-R (among other tests) online during the sign-up stage of recruitment. Our procedure took approximately 20 min to complete, and participants were also asked a set of additional questions for publication elsewhere (concerning synaesthesia).

## Results

Since some participants completed different elements of our tasks (e.g. because they left before the end of the study), we preface our results with the number of participants in each test. All data here and throughout approximated normal distributions and so parametric statistics were used. We conducted a series of ANOVA’s to investigate group differences in each of our measures separately.

### Autism Spectrum Quotient

**Fig. 2:**
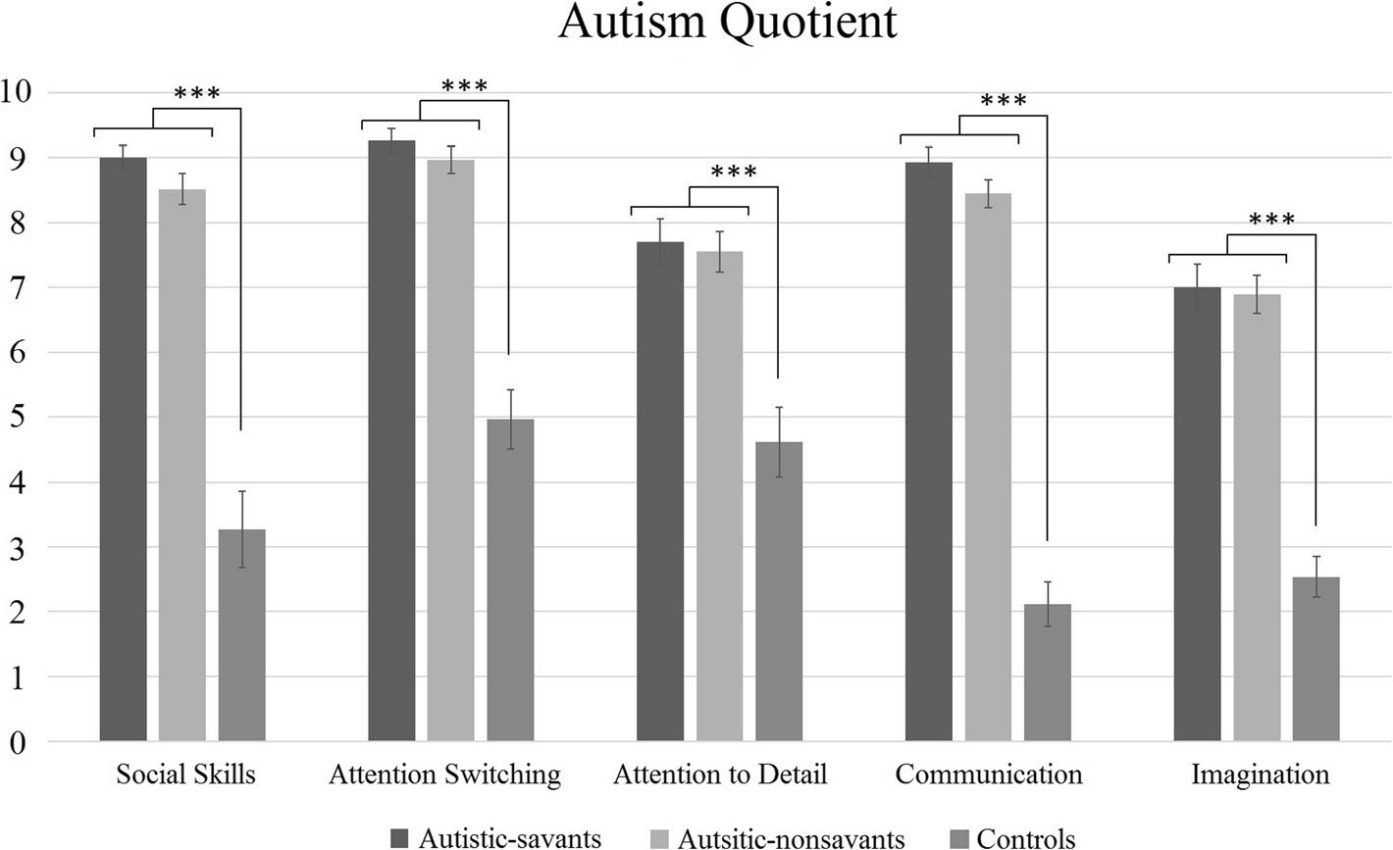
The profile of AQ scores by factor and group scores (error bars show SEM). Asterisks here and throughout indicate significance at *p < .05; **p < .01; ***p < .001

AQ data was collected from 33 autistic-savants, 30 autistic-nonsavants, and 28 controls, and Fig. 2 shows every factor of the AQ. We conducted a 3 × 5 ANOVA contrasting group (autistic-savants, autistic-nonsavants, controls) and the individual AQ factors (social skills, attention switching, attention-to-detail, communication, imagination), and a main effect of group was found (*F*(2, 88) = 96.96, *p* < .001, ηp2 = .69). There was also a main effect of factor (*F*(4, 352) = 29.50, *p* < .001, ηp2 = .25) and an interaction between group and factor (*F*(8, 352) = 7.44, *p* < .001, ηp2 = .15). Post hoc comparisons with Bonferroni correction revealed the same pattern of results for every factor, that is, a significant difference between autistic-savants and controls (all *p* < .001) and between autistic-nonsavants and controls (all *p* < .001), but not between autistic-savants and autistic-nonsavants (all *p* > .05).

Where we found null results between autistic-savants and autistic-nonsavants for the AQ, we calculated Bayes factors to determine whether null results indicated no difference or a lack of statistical power.^1^ We selected an informed prior (i.e. the mean difference we might expect between our participant groups, and its standard error) from an earlier study [43] using the same dependent variable as the current study. This prior was generated by looking at the difference in AQ scores between UK Mathematics Olympiad winners (*N* = 16) and autistic individuals (*N* = 58), and we treat Mathematics Olympiad Winners as a comparable group to autistic-savants in our own study (i.e. both groups display some form of exceptional skill). This comparison was chosen because we are looking to see whether differences truly exist between our autistic-savants and autistic-nonsavants. Our Bayes factors suggested support for the null hypothesis (i.e. no differences between groups) for four of the five AQ factors (social skills BF < .33; communication BF < .33; attention switching BF < .33; imagination BF = .35) with the exception of attention-to-detail, for which no firm conclusions could be drawn (BF = 0.96). Refer to Additional file 1 for more information regarding our calculation of the above Bayes factors including our choice of parameters as well as a sensitivity analysis.

### Systemising Quotient-Revised

**Fig. 3:**
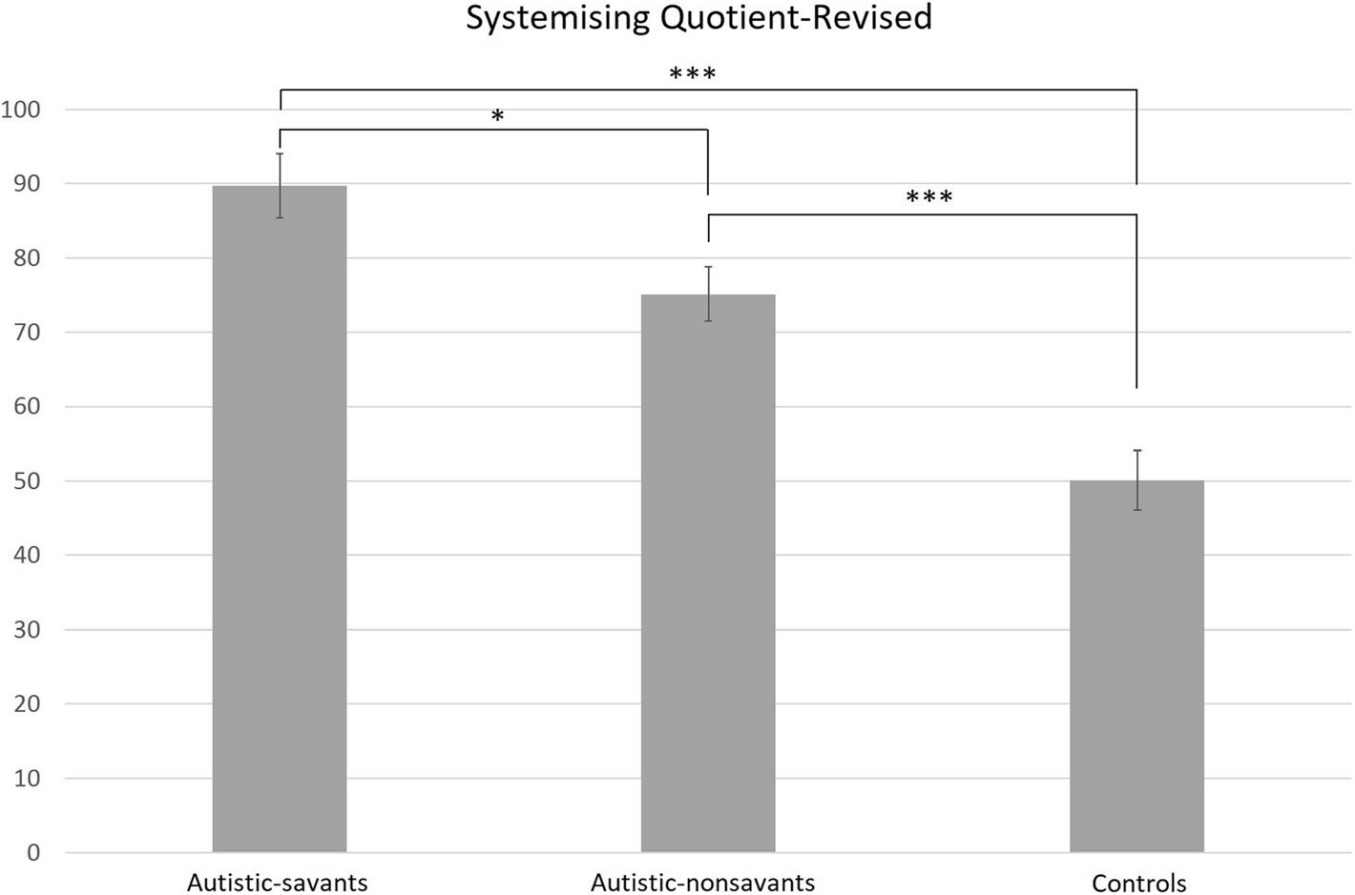
Group differences in mean SQ-R scores (error bars show SEM)

SQ-R data was collected for 31 autistic-savants, 33 autistic-nonsavants, and 27 controls, and their data is shown in Fig. 3. A one-way ANOVA comparing these differences revealed a significant main effect, *F*(2, 90) = 23.94, *p* < .001, ηp2 = .35. Post hoc comparisons with Bonferroni correction revealed significant differences between the autistic-savant and autistic-nonsavant group (*p* = .022), the autistic-savant and control group (*p <* .001), and the autistic-nonsavant and control group (*p <* .001). In other words, the pattern was autistic-savants > autistic-nonsavants > controls.

### Glasgow Sensory Questionnaire

**Fig. 4:**
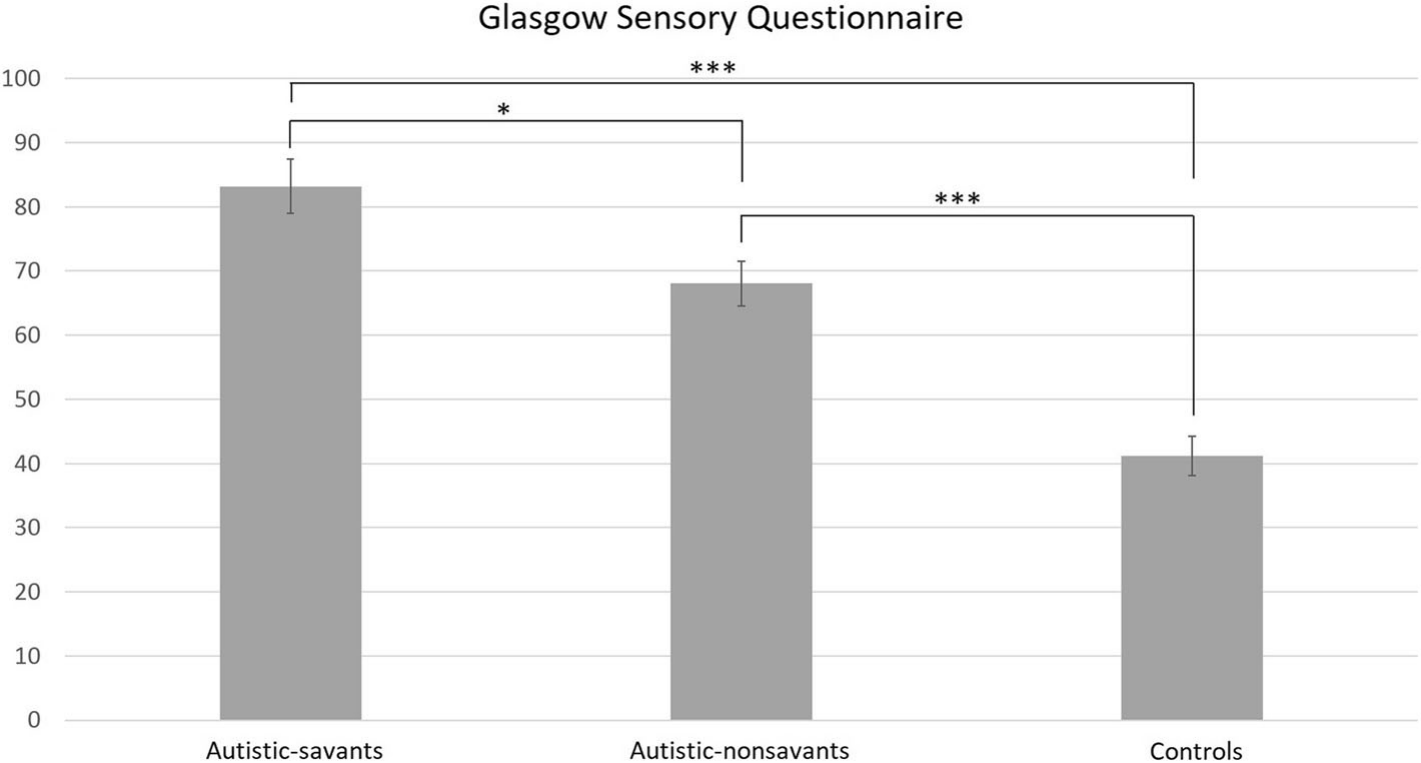
Group differences in mean GSQ score (error bars show SEM)

All participants completed this test. Figure 4 displays participants’ total GSQ scores for the autistic-savant, autistic-nonsavant, and control group. A one-way ANOVA comparing these differences revealed a significant main effect, *F*(2, 110) = 29.35, *p <* .001, ηp2 = .35. Post hoc comparisons with Bonferroni correction revealed significant differences in total GSQ scores between the autistic-savant and autistic-nonsavant group (*p* = .030), the autistic-savant and control group (*p <* .001), and the autistic-nonsavant and control group (*p <* .001). In other words, the pattern again was autistic-savants > autistic-nonsavants > controls.

### Sussex Cognitive Styles Questionnaire

**Fig. 5:**
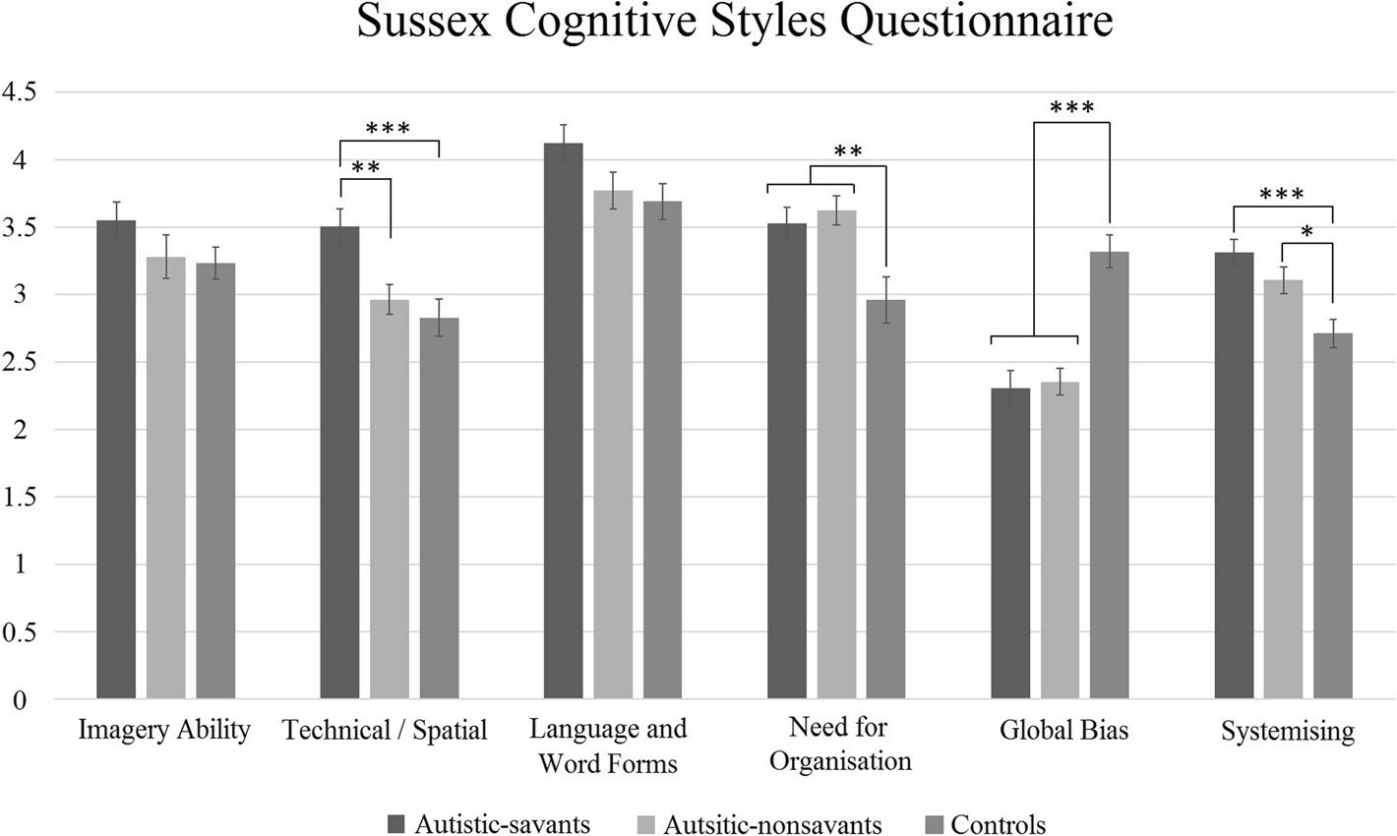
The profile of SCSQ scores by factor and group (error bars show SEM)

All participants completed this test. Figure 5 shows all factors of the SCSQ. We conducted a 3 × 6 ANOVA contrasting group (autistic-savants, autistic-nonsavants, controls) and the individual SCSQ factors. We found a significant main effect of group (*F*(2, 108) = 6.06, *p* = .003, ηp2 = .10), a significant main effect of factor (*F*(5, 540) = 31.84, *p* < .001, ηp2 = .23), and an interaction between group and factor (*F*(10, 540) = 7.69, *p* < .001, ηp2 = .13).

Post hoc comparisons with Bonferroni correction revealed significant differences (all *p* < .05) between autistic-savants and controls on technical/ spatial, need for organisation, global bias, and systemising. Significant differences (all *p* < .05) were also found between autistic-nonsavants and controls on need for organisation, global bias, and systemising. A significant difference was also found between autistic-savants and autistic-nonsavants on technical/spatial (*p* = .005). No significant differences were found between any group for ‘imagery ability’ or ‘language and word forms’. As before, we calculated Bayes factors to determine whether these null results indicated no difference or a lack of statistical power. This time, however, no suitable previous studies exist from which to draw informed priors. We therefore used an uninformative prior with the H1 (prior distribution) modelled as a uniform distribution in which all effects within a specified interval are considered equally likely (given no previous evidence to inform our decision). Following the standard procedure, we entered the lowest and highest possible mean differences between groups (i.e. zero and [maximum score per factor minus minimum score] respectively). Our calculation of Bayes factors suggests evidence for the null hypothesis for both imagery (BF = .22) and language (BF = .30). In summary, we found that autistic individuals, irrespective of savant syndrome, scored higher than controls on need for organisation, systemising, and local bias (i.e. low global bias). In addition, autistic-savants out-performed controls and autistic-nonsavants in technical/spatial traits.

### Leyton Obsessional Inventory—short form

**Fig. 6:**
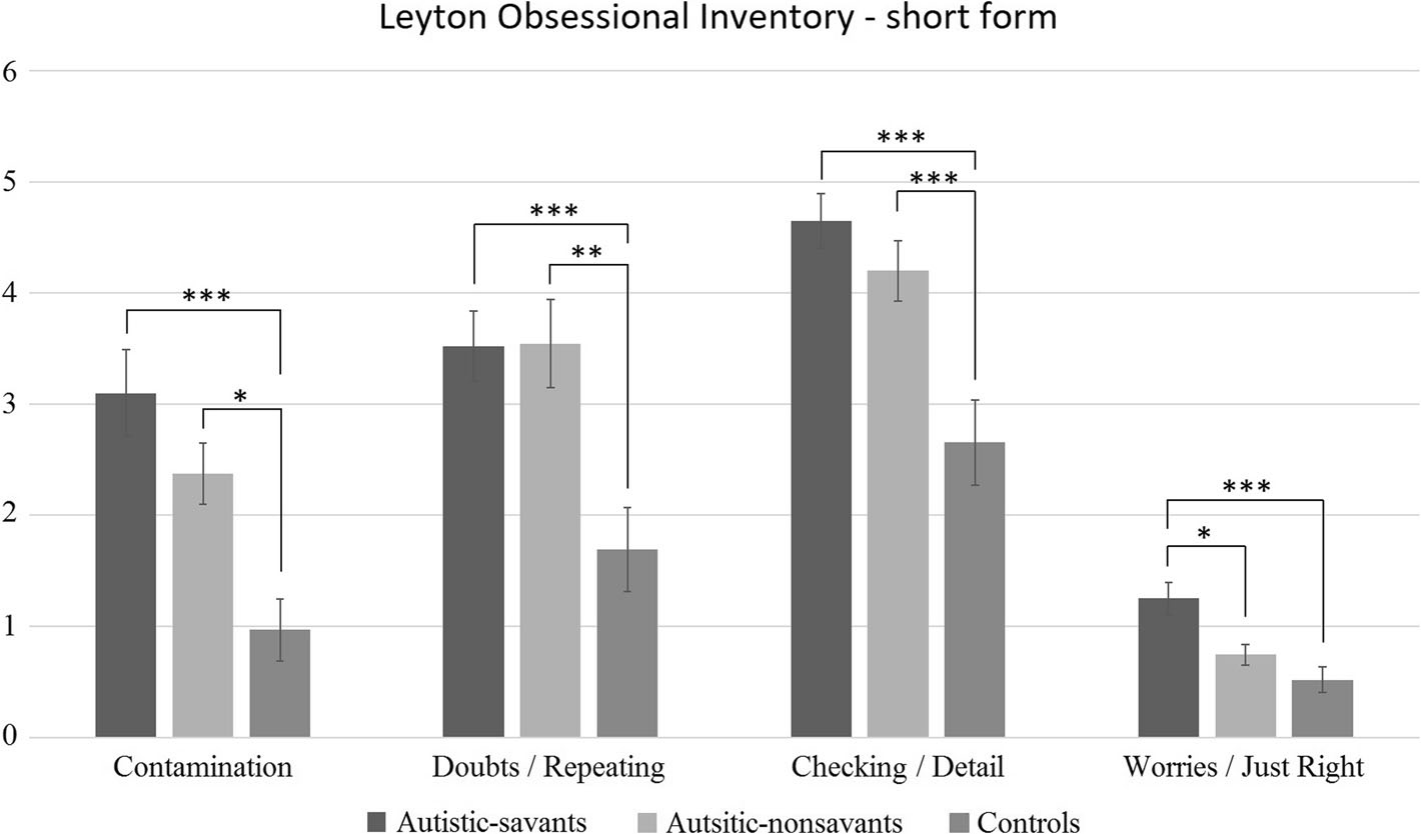
The profile of LOI scores by factor and group (error bars show SEM)

All participants completed this test. Figure 6 shows all factors of the LOI across groups. We conducted a 3 × 4 ANOVA contrasting group (autistic-savants, autistic-nonsavants, controls) and the individual LOI factors (contamination, doubts/repeating, checking/detail, worries/just right). There was a significant main effect of group (*F*(2, 108) = 16.28, *p* < .001, ηp2 = .23), a significant main effect of factor (*F*(3, 324) = 90.78, *p* < .001, ηp2 = .46), and a significant interaction (*F*(6, 324) = 2.85, *p* = .01, ηp2 = .05).

Post hoc comparisons with Bonferroni correction revealed significant differences between autistic-savants and controls on every factor (all *p* < .05). Significant differences were also found between autistic-nonsavants and controls on every factor (all *p* < .05) apart from the worries/just right factor (*p* = .58). Finally, a significant difference between autistic groups emerged on the worries/just right factor with autistic-savants scoring higher than autistic-nonsavants (*p* = .02).

We also found that seven autistic-savants as well as two autistic-nonsavants and one control scored above the threshold of a score of 20 or more which suggests obsessive-compulsive disorder (OCD) symptoms. However, a chi-square test of association between the rates of OCD symptoms in the three groups did not reach significance (*χ*^2^(2) = 4.34, *p* = .11.

### Sussex Savant Questionnaire

All participants completed this test, whose aim had been to separate our autism sample into our two autism sub-groups (autistic-savants and autistic-nonsavants). Table 1 shows the categories of skills asked about during the study along with the number of cases of each skill reported by participants. For completeness, the Additional file 2 contain descriptive statistics for the various sub-scales of our above questionnaire measures broken down according to the presence or absence of particular savant skills, but we do not consider them in detail here due to the large number of measures and lack of power when smaller samples are divided in this way.

**Table 1:** Types of savant skills reported by the autistic-savant group, some participants reported having multiple savant skills

Skill types	Number of cases
Math	16
Calendar calculation	3
Musical instrument playing	6
Music reproduction	9
Absolute pitch	12
Art	16
Memory	26
Mechanical (building)	8
Fluency for different languages	12
Other	25

As an additional validation of our methodology, we looked again at the skills reported in Table 1, to see whether these self-reports could be directly tied to our measures. We found a ‘dose-like’ effect in the number of savant skills reported within our savant group. Here a significant correlation was found between the number of savant skills reported and the strength of the technical-spatial abilities found in our *Sussex Cognitive Styles Questionnaire* (*r* = .43, *p*_corrected_ = .01); none of our other above effects were significant (all *p*’s_corrected_ > .05). Finally, we note that there were gender imbalances across our groups (see [46] for gender effects in autism). For an exploration of the effects of gender on all of our above measures, see footnote.^2^

## Discussion

Our results reveal a distinct profile of group differences between autistic-savants and autistic-nonsavants. The autistic-savants differed from autistic-nonsavants in that the former had heightened sensory sensitivity, greater obsessional behaviours (relating to excessive worries and getting things ‘just right’), more systemising traits, and increased technical/spatial traits (i.e. technical interests, mathematical abilities, and the use of spatial mental imagery). In all instances, these traits are features of autism more generally (i.e. they also discriminated between autistic-nonsavants and controls) but were particularly enhanced in savant syndrome specifically (i.e. discriminating autistic-savants from autistic-nonsavants). However, it is not the case that savants are simply shifted upwards along the autism spectrum. We did not find any differences between autistic-savants and autistic-nonsavants on the AQ or on subscales relating to attention-to-detail or social and communication skills, which might otherwise have been expected based on previous theoretical accounts [15, 23]. The implications of these findings for other theoretical models are discussed in more depth in the ‘General discussion’ section.

## Experiment 2: learning the novel savant skill of calendar calculation

The purpose of experiment 2 was to explore whether participants could be trained to perform a characteristic savant skill—calendar calculation—and to investigate whether autistic-savants would show differences in accuracy or learning-style compared to autistic-nonsavants. As before, controls without autism or savant skills were included to separate effects linked to autism from effects linked to savant syndrome. Participants learned a number of different calendar rules throughout a training session and were given a final test that tapped all the rules. For example, the ‘matching month’ rule states that within any non-leap year, certain months have matching structures (January = October; March = November = February; September = December; July = April; e.g. if 1 March is a Sunday, then it necessarily follows that 1 November and 1 February will also be Sundays in that year). Savants who have calendar calculating within their repertoire are already sensitive to these rules [47]. For instance, they are faster at saying that 1 November is Sunday if it has been ‘primed’ by a preceding question about 1 March (which has the same answer, as its ‘matching month’) than if preceded by 1 September (which has a different answer). As well as examining the overall ability to learn the task, we can use this pattern of response times (i.e. faster responses for primed answers) as a measure of the degree to which the rules have been internalised and are utilised by all subjects, and furthermore, whether savants perform differently in either accuracy or speed.

In summary, this study aimed to determine whether people with savant skills have a natural aptitude for learning this kind of information or whether they approach the task with different strategies. If so, we assess whether this is linked to autism per se or linked only to those autism subjects with pre-existing savant abilities (excluding calendar calculation). We predict that savants may show either a superior level of accuracy or a different style of approach to the question (this latter suggested by response time measures and/or a post hoc questionnaire).

### Participants

Fifty-eight participants took part in experiment 2, 14 of whom also took part in experiment 1 above (6 autistic-savants, 6 autistic-nonsavants, and 2 controls). The participants comprised 13 autistic-savants (4 female; mean age 37.54, range 23–56, SD = 9.11), 10 autistic-nonsavants (5 female; mean age 39.20, range 27–51, SD = 9.02), and 35 controls (29 female; mean age 32.26, range 20–50, SD = 11.21). A one-way ANOVA showed no significant differences between groups on age, *F*(2, 57) = 2.37, *p =* .10, or highest qualification, *F*(2, 57) = 2.23, *p =* .12. All individuals in the autism groups (autistic-savant; autistic-nonsavant) self-reported having a formal diagnosis of autism in our questionnaire (see the ‘Procedure’ section): 3 autism, 18 Asperger syndrome, and 2 other. All autistic-savants, and no other group, self-reported having a savant skill.

Participants were recruited from two sources. Forty-two participants were recruited from CARD (13 autistic-savants, 10 autistic-nonsavants, 19 controls). The remaining 16 participants (all controls) were recruited from the University of Sussex community. Participants were entered into a £50 prize-draw for their participation, and our study was approved through the Cross-Schools Science and Technology Research Ethics Committee at the University of Sussex. In addition to the above participants, a further 22 were initially recruited but later excluded. These were 13 participants who used incorrect response buttons (i.e. the right-hand numeric keypad rather than the number keys) and 9 participants who were not engaging in the task. Three of these had response times that were not within a feasible range (i.e. < 700 ms; given the mean average RT for other subjects of 12.4 s; SD = 5.3) and 6 scored below chance, indicating they had not engaged with the calendar rules presented during our test.

### Materials and procedure

All participants received an initial email invitation and accessed the study by clicking on a link embedded in the email that took them to the information and consent page. Participants then gave demographic information and next completed the Sussex Savant Questionnaire (SSQ) in the same way as in experiment 1 above. Participants then completed additional questionnaires to be published elsewhere (involving synaesthesia). All participants then completed a test of mental arithmetic (henceforth ‘maths test’) to ensure there were no a priori differences across groups in maths ability. In this, participants saw 20 questions requiring the addition of a pair of two-digit numbers (e.g. 76 + 43). Participants were required to calculate the answer as quickly as possible and type it into the box provided. Following the maths test, participants then began their calendar calculation training.

The calendar calculation training took place entirely online using Inquisit, an online experiment-hosting software and lasted around 35 min. Participants completed a training session (composed of three tutorials) followed by a final test at the end of the session. Each tutorial explained a set of patterns and calendar rules that can be used to calculate days of the week for certain dates. Tutorial 1 taught participants about the *matching-month rule* that explains that certain months cluster into groups regarding their weekdays (see above for a further explanation). Tutorial 2 taught participants the *follow-on month rule* which states that months of the year can be arranged in a particular sequence to calculate days of the week faster (e.g. if 1 March 2015 is a Sunday, then it follows that 1 June is a Monday and 1 September is a Tuesday). Tutorial 3 focused on the *1-8-15-22-29 rule* which states that the 1st, 8th, 15th, 22nd, and 29th days of the month all fall on the same day of the week (e.g. in March 2015 all these dates fell on a Sunday). Each tutorial was accompanied by examples of calendar images to aid learning. At the end of each tutorial, participants were given 2 min to memorise the material just covered (without writing anything down) and then answered a set of tutorial questions based on those rules. At the end of all three tutorials, they completed the final calendar calculation test (see below).

For the purposes of this study, we focused only on teaching participants how to calculate days of the week for the year 2015 (due to the time limitations of a single study session). All questions (tutorial and final test) were forced choice with each answer being one of the seven days of the week. Participants answered using keys 1–7 on the keyboard and were given feedback (‘correct’; or what the correct answer should have been e.g. ‘Tuesday’). During the very first tutorial, participants with incorrect responses had to then select the correct answer to continue.

After all tutorials, participants completed the final calendar calculation test. The test contained 40 questions that spanned all the rules that had been taught previously and which again were dates that required participants to supply their weekday. Within these questions, there were 20 ‘primed’ and 20 ‘unprimed’ dates. Primed dates could be answered more easily than un-primed dates by reference to the question before, given the rule of ‘matching months’. As noted above, this rule exploits the fact that 2015 has four groups of months, such that dates within each group fall on the same weekday (e.g. January and October are within the same group, so 8 January will fall on the same weekday at 8 October). Hence, ‘primed’ questions should be easier to answer because the response is the same as the question before (e.g. What weekday was 8 January? Answer: *Thursday*; PRIMED = What weekday was 8 October 2015? Answer: *Thursday*; UNPRIMED = What weekday was 8 November 2015? Answer: *Sunday*).

After the test, participants completed a questionnaire (see Additional file 2) with two sub-sections, asking how much they had enjoyed the study (Q7, Q8, Q9) and what strategies they used (Q1, Q2, Q3, Q4). These questions were presented on a 1–5 Likert scale (strongly disagree, disagree, neither agree nor disagree, agree, strongly agree). An additional question (Q5) was to ensure subjects were paying attention, and two final optional questions provided text boxes to enable participants to add further information if they wished (Q6 and Q10; not analysed). Once this enjoyment/strategy questionnaire was complete, participants saw a final screen thanking them for their time.

## Results

### Sussex Savant Questionnaire

Table 2 shows the categories of skills asked about during the study. Importantly, no autistic-savants reported calendar calculation as one of their savant skills, meaning they should not have an advantage to other groups based on prior abilities.

**Table 2:** Types of savant skills reported by the autistic-savant group in experiment 2, some participants reported having multiple savant skills

Skill types	Number of cases
Math	5
Calendar calculation	0
Musical instrument playing	2
Music reproduction	2
Absolute pitch	4
Art	2
Memory	5
Mechanical (building)	1
Fluency for different languages	1
Other	5

### Maths pre-test

There were no significant differences in mental arithmetic accuracy between the autistic-savants (*M* = 19.36, SD = .51), autistic-nonsavants (*M* = 19.1, SD = 1.29), and controls (*M* = 19.34, SD = 1.06), *F*(2, 55) = .76, *p* = .475. There were also no significant differences in response times between the autistic-savants (*M* = 6899, SD = 1887), autistic-nonsavants (*M* = 7675, SD = 1888), and controls (*M* = 7100, SD = 2200), *F*(2, 55) = .420, *p* = .659. This means that all things considered, no group started with any a priori maths advantage.

### Calendar calculation test

For accuracy scores, we conducted a 3 × 2 ANOVA contrasting group (autistic-savants, autistic-nonsavants, controls) and question type (primed vs. unprimed questions). As expected, we found a significant main effect of question type (*F*(1, 55) = 26.82, *p* < .001, ηp2 = .33) such that scores were higher for the easier primed questions (*M* = 14.85, SD = 5.15) compared to unprimed questions (*M* = 12.97, SD = 5.98). This suggests that participants were applying rules appropriately in our task and paying attention. We also found a statistical trend for a main effect of group (*F*(2, 55) = 2.56, *p* = .09, ηp2 = .09), with controls (*M* = 15.44, SD = 6.04) tending to have overall higher accuracy scores compared to the autistic-nonsavants (*M* = 11.60, SD = 11.30; *p* = .084), but not compared to the autistic-savants (*M* = 14.73, SD = 9.92; *p* = 1.00). Finally, there was no significant interaction between group and question type (*F*(2, 55) = 1.96, *p* = .15, ηp2 = .07).

**Fig. 7:**
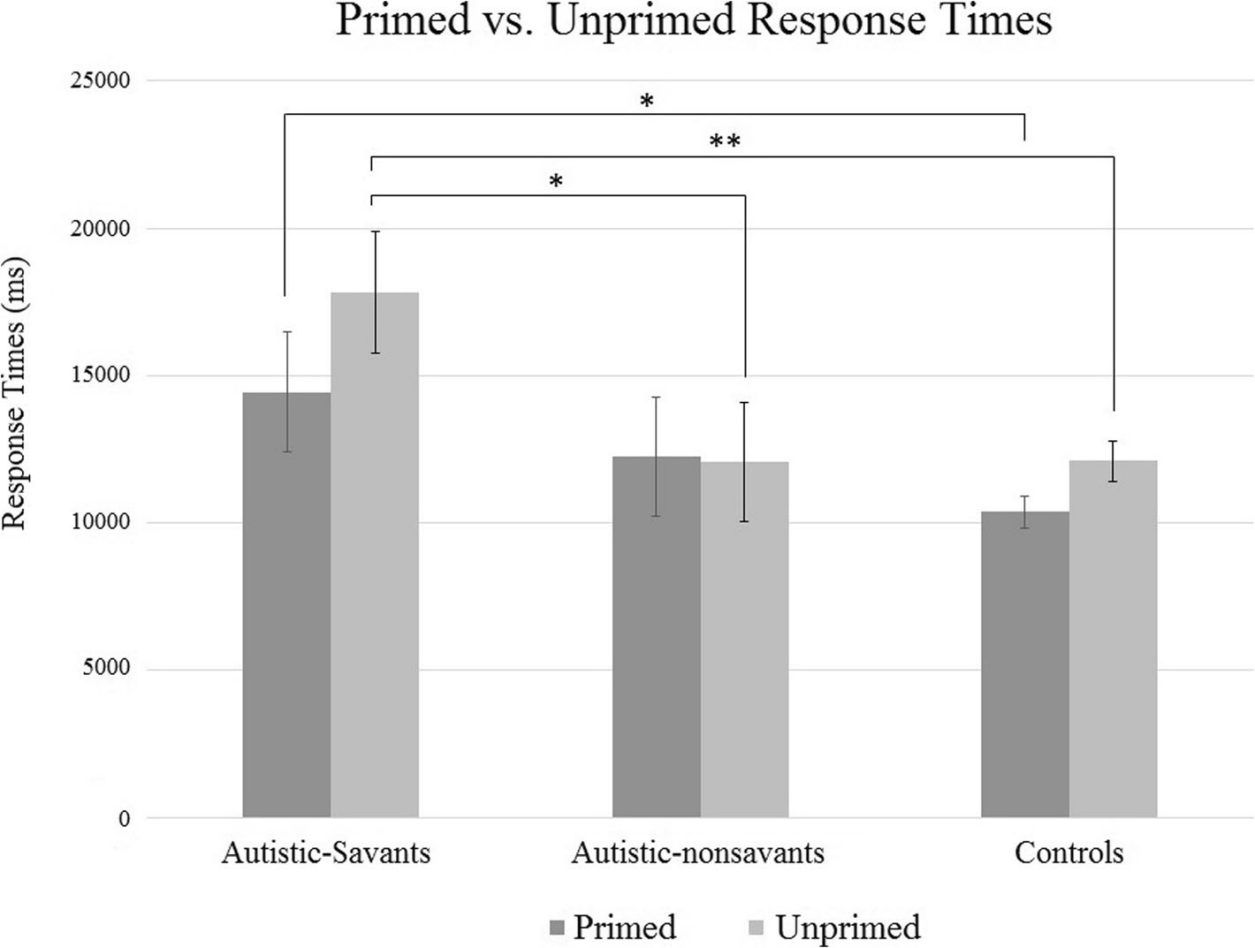
Response times for the primed and unprimed dates between groups (error bars show SEM)

We also conducted a 3 × 2 ANOVA (again, group × question) looking at participants’ response times. We again found a significant main effect of question type (*F*(1, 55) = 16.78, *p* < .001, ηp2 = .23) such that participants were significantly faster for the easier primed questions (*M* = 12,351, SD = 5703) compared to unprimed questions, as expected (*M* = 13,994, SD = 6241). Importantly, we found a significant main effect of group (*F*(2, 55) = 4.55, *p* = .015, ηp2 = .14) and a significant interaction between group and question type (*F*(2, 55) = 5.12, *p* = .009, ηp2 = .07). Detailed explorations revealed that autistic-savants (*M* = 17,832, SD = 7500) were significantly slower on the unprimed questions (Fig. 7) compared to both autistic-nonsavants (*M* = 12,055, SD = 6352; *p* = .043) and controls (*M* = 12,094, SD = 4129; *p* = .006), and autistic-savants (*M* = 14,447, SD = 7325) were significantly slower than controls even on the primed questions (*M* = 10,371, SD = 3148; *p* = .043).

### Enjoyment/strategy questionnaire

A one-way ANOVA found no significant differences (*F*(2, 53) = 1.41, *p* = .25) in how much each group enjoyed learning to calendar calculate (i.e. collapsing questions Q7, Q8, Q9): for autistic-savants (*M* = 3.44, SD = .88), autistic-nonsavants (*M* = 2.89, SD = 1.17), or controls (*M* = 3.42, SD = .77).

In terms of strategy used, we conducted a 3 × 4 ANOVA crossing group (autistic-savant; autistic-nonsavant; control) and strategy question (Q1, Q2, Q3, Q4; relating respectively to picturing a mental calendar; using the on-screen timeline *Mon*, *Tues*, *Wed*…; using mental arithmetic; using rote memorisation of anchor dates). We found no main effect of group (*F*(2, 51) = 1.77, *p* = .180, ηp2 = .07) and no interaction (*F*(6, 153) = .93, *p* = .476, ηp2 = .35). But we found a significant effect of question (*F*(3, 153) = 9.43, *p* < .001, ηp2 = .16) in that the strategy of ‘picturing a calendar in my head’ was used least often compared to the other three strategies (all *p <* .05). No other comparisons were significant (all *p* > .05).

## Discussion

The results for experiment 2 showed no clear a priori group advantages in being able to learn to perform calendar calculation skills. However, a significant pattern emerged for response times in that autistic-savants were slower than both autistic-nonsavants and controls, when tackling the harder unprimed date questions. They were also slower than controls even in the simpler primed questions. This suggests that autistic-savants engaged with the task in a distinct way compared to the other groups in that they take longer to respond. We also found that the least-used strategy was ‘picturing a calendar in my head’ but that all groups reported similar strategies and enjoyed the task to a similar degree. The implications of these results are discussed below.

### General discussion

The purpose of these studies was to profile the differences between autistic participants with and without a prodigious talent (autistic-savants and autistic-nonsavants, respectively). The third group was controls with neither autism nor a prodigious talent. Our findings present the first empirical evidence to adjudicate between different theoretical frameworks of savant syndrome in adults. Each of our results is discussed in turn below in terms of how they relate to previous models of the development of savant skills.

Experiment 1 investigated the profile of self-reported differences between autistic-savants, autistic-nonsavants, and controls. We asked all groups to complete self-report measures from six questionnaires: the Sussex Savant Questionnaire (SSQ), the Glasgow Sensory Questionnaire (GSQ) [30], the Leyton Obsessional Inventory—short form (LOI) [40], the Sussex Cognitive Styles Questionnaire (SCSQ) [41], the Systemising Quotient-Revised (SQ-R) [42], and the Autism Spectrum Quotient (AQ) [43]. Our aim was to establish a general profile of individual differences that might distinguish between autistic individuals who develop talent and autistic individuals who do not. Our choice of questionnaires was motivated by previous theories and findings [3, 15, 16, 23, 24], and we focused on factors related to sensory sensitivity, obsessive behaviours, different aspects of cognitive style (e.g. local bias), and autism-related traits such as systemising and social awareness. We first briefly describe the (expected) pattern of results that distinguished all participants with autism from controls.

We found that both autism groups (autistic-savants and autistic-nonsavants) differed from controls on key measures, as predicted from previous literature [30, 43, 46] and theoretical accounts [3]. Both autistic-savants and autistic-nonsavants, relative to controls, reported more symptoms related to sensory sensitivity (known previously to be heightened in autism [30]) and obsessive behaviours (a common hallmark of autism [2]), increased systemising (previously shown in autism [46]), and a more locally oriented cognitive style (theorised as a feature of autism and savant syndrome and supported by findings [3, 25] but savant syndrome had not been separated from autism). Both autism groups also reported the expected generalised autism-related symptoms such as poor social, communication, and imagination skills, as well as poor attention switching and heightened attention-to-detail, which replicates previous findings using the same self-report measure in autism [43]. These findings are useful in confirming the validity of our autism classifications (autistic-savant and autistic-nonsavant) against controls and suggest that savant syndrome does indeed exist within or alongside autism based on our measures.

Our key findings relate to differences between autistic-savants and autistic-nonsavants. We found that these two groups differed in several ways. First, we considered two models that theorise why savants engage in many hours of practice [15, 16]. Happé and Vital’s [15] mind-blindness theory suggests that autistic-savants practice as a result of re-dedicating cognitive resources to skill development that would otherwise be used to monitor social interactions. This predicts that autistic-savants may show poorer social skills compared to autistic-nonsavants. Our findings did not support this hypothesis: there were no differences between autistic-savants and autistic-nonsavants on social or communication skills in the AQ (and indeed no difference in any subscale of the AQ). Since it would have been expected that autistic-savants would score higher than autistic-nonsavants on generalised autism-related symptoms, we additionally showed that a Bayes factor analysis supported the null hypothesis of no differences between these groups for four out of the five AQ sub-scales (social skills, attention switching, communication, imagination). This does not necessarily rule out altogether the role of additional autism-related traits in the development of savant skills (e.g. a preference for solitary activities), but our current data suggests that differences on the above measures may not be strongly apparent when comparing autistic-savants and autistic-nonsavants.

Instead, we found support for an alternative model by Simner and colleagues [16] in which practice arises from increased obsessional traits in autistic-savants. The autistic-savant group showed higher obsessional traits compared to autistic-nonsavants, and this was specifically related to the ‘worries/just right’ factor. This factor relates to the inclination to take one’s time about making sure things are ‘just right’ (e.g. ‘I do not take a long time to dress in the morning’ [reverse coded]). This factor could well be implicated in the development of talent, for example, when making sure the details of a painting are ‘just right’ or putting additional effort into learning a number list perfectly without error. The second feature of the ‘worries/just right’ factor (i.e. excessive worries about, e.g. bumping into people or the belief in unlucky numbers) raises an interesting possibility that obsessive rehearsal in savants might be driven by anxiety. If so, then savant skills may be guided by the same anxiety-laden motivations that drive, for example, repetitive OCD behaviour [19]. Indeed, seven autistic-savants (compared to two autistic-nonsavants and one control) scored above the threshold for OCD symptoms although our small numbers did not allow us to support this statistically. We are therefore exploring in subsequent studies how anxiety may be implicated in the development of savant skills. Overall, the above results suggest that practice in savant skills is driven by obsessional (possibly anxiety-linked) behaviours in autistic-savants compared to autistic-nonsavants [16] rather than freed-up resources from mind-blindness [15].

We also investigated other areas of cognition/perception, drawn from several theoretical accounts [3, 23, 24]. We found that autistic-savants scored higher on the Systemising Quotient-Revised (SQ-R; although not on the shorter ‘systemising’ factor of the Cognitive Styles Questionnaire; SCSQ). We also found that autistic-savants scored higher on ‘technical/ spatial’ elements of the SCSQ which relates to technical interests, mathematical ability, and the use of spatial mental imagery—but also contains several questions which are systemising in nature (e.g. ‘If I were buying a stereo, I would want to know about its precise technical features.’). Together, these findings of higher systemising and technical/spatial abilities of savants support the model by Baron-Cohen et al. [23] who proposed that savant skills emerge from increased systemising in autistic-savants. Where we found null results between all group comparisons, we additionally computed Bayes factors to assess whether our results truly reflected no differences. Here our analysis supported evidence for the null hypothesis of no differences between autistic-savants and autistic-nonsavants on the imagery ability and language and word forms sub-scales of the SCSQ; therefore, our current data suggest that these aspects of cognition may not be involved in the facilitation of savant skills.

Local processing has also been theorised as important in the development of savant skills, as suggested by the enhanced perceptual functioning model (EPF) [3]. However, we found no difference in self-reported local processing traits between autistic-savants and autistic-nonsavants, and so fail to support this proposal from the current data. Bennet and Heaton [22] found a similar pattern to us in savant children and adolescents based on parental reports (no local processing advantage for autistic-savants over autistic-nonsavants). Importantly, however, Pring et al. [26] show that enhanced local processing abilities in savants (relative to autistic-nonsavants) might only be revealed by a more engaging task. As such, the EPF model by Mottron et al. [3] has been supported by behavioural evidence in certain engaging tasks, but not by our self-report data here.

Finally, we investigated the theory that the development of savant skills might be tied to heightened sensory sensitivity [23]. Our autistic-savants reported significantly more symptoms related to sensory sensitivity lending support to the theory that sensory sensitivity could act as an initial catalyst in the emergence of savant talent. Baron-Cohen et al. also made claims that sensory sensitivity might increase attention-to-detail. However, although we found this trait to be heightened in our autism groups globally, there was no difference in attention-to-detail between our autistic-savants and autistic-nonsavants. Having said this, our Bayes analysis suggested that no firm conclusions could be drawn about group differences in attention to detail; therefore, future studies may wish to further investigate this. Interestingly, the finding of heightened sensory sensitivity in our savant group relates more broadly to another condition, synaesthesia, which also has a distinct sensory component. As noted in the Background, synaesthesia produces sensory experiences that are induced by unusual stimuli (e.g. letters or numbers might induce colour sensations). Synaesthesia has been linked to autism previously [48, 49], and Ward et al. [50] showed that both conditions share common links in their profile of sensory sensitivities. More recently, synaesthesia has been specifically tied to savant syndrome rather than autism per se [39]. So our current data combined with previous evidence further suggests that sensory components may be an important mediating link between autism and the development of savant skills, perhaps even via synaesthesia itself [16].

In experiment 2, we taught the three groups the novel skill of calendar calculation and tested their abilities after three tutorials. We aimed to examine whether autistic-savants would show advantages in learning this skill compared to autistic-nonsavants and controls, as predicted by the ‘veridical mapping’ model [24]. Veridical mapping links savant talent to an enhanced ability to detect regularities within and between systems. Calendar calculation requires this skill *par excellence* because weekdays can be mapped to dates by understanding the underlying regularities in the calendar—which we taught to the participants in our study. In contrast to veridical mapping, practice-based models of savant skills might not predict immediate advantages prior to prolonged training [15, 16]. We found no evidence to support the veridical mapping model since autistic-savants were no more accurate than autistic-nonsavants or even controls. It is possible that differences in accuracy may have been observed if participants were given a longer period of training, for instance, if autistic-savants were given more time to consolidate their learning. Indeed, calendar calculation is often assumed to develop as a result of periods of study which are far longer than our training session. However, we show that calendar calculation is surprisingly easy to acquire with around 75% accuracy after merely 35 min of training even in the control group.

Importantly, we did find that autistic-savants took significantly longer to answer our calendar calculation questions: they were slower than both autistic-nonsavants and controls for (difficult) unprimed questions and slower than controls even on (easier) primed questions. One interpretation of this is that our autistic-savant participants may have found the task more difficult compared to the other groups. But given that all subjects began with the same level of mental maths ability (as measure by our test in experiment 2), a more plausible interpretation is that, autistic-savants engaged with the task differently by adopting a more careful, effortful approach with increased checking. This would fall in line with the findings from experiment 1 that autistic-savants show more obsessional behaviours, specifically related to taking a long time to get things ‘just right’ (see results above for the Leyton Obsessional Inventory). Indeed, the magnitude of the differences between groups for response times (autistic-savants took more than 5 s longer on average than nonsavants) suggests again they may have taken longer to check and re-check their answers. Overall, experiment 2 lends support to practice-based models of savant skills rather than veridical mapping since autistic-savants did not show immediate advantages on this skill prior to extended training and they appear to display a more engaged, effortful approach to the task.

One limitation of the current study is that we validated savants with a detailed self-report questionnaire rather than by objective tests. This is largely because savant syndrome is an umbrella term for many different heterogeneous manifestations (e.g. calendar- calculation, drawing, music etc.). We did however validate our approach by showing a ‘dose-like’ effect of savant skills on one of our other measures: the number of savant skills reported in our questionnaire correlated positively with the strength of savants’ technical-spatial abilities. In other words, although talents are described only in self-report (rather than objectively evaluated), this self-report appears to be a reliable metric since it correlates with a trait that particularly separates autistic-savants from autistic-nonsavants. Nevertheless, future investigations might focus on objectively verifying self-reported skills with a battery of tests designed to measure specific savant skills (e.g. absolute pitch, language skills), and we have embarked on this program of research in our own lab. A further limitation of our study was the fact that we had a high proportion of females in the control group compared to our two autism groups. Nonetheless, we conducted an additional analysis where we had found main effects (i.e. sensory sensitivity, obsessional traits, technical-spatial skills, and systemising) showing that our pattern of results was maintained across all groups even after controlling for gender.

## Conclusions

Our results demonstrate a diverse range of attributes that distinguish autistic-savants from autistic-nonsavants in adults based on both self-report and an objective test. Our findings suggest that savant syndrome is defined by observable differences in aspects of cognition, perception, and behaviour that go beyond the mere presence of savant skills themselves. We found that areas of particular influence on savant talent relate specifically to higher sensory sensitivity (supporting Baron-Cohen et al. [23]), obsessive behaviour (supporting, e.g. Simner et al. [16]), and systemising and technical/spatial traits (supporting Baron-Cohen et al. [23]) along with a more careful and engaged learning style when presented with a novel savant skill (supporting practice models such as Simner et al. [16]). We did not find social skills [15], local processing [3], or increased pattern detection in calendar-calculation [24] to be distinguishing features between autistic-savants and autistic-nonsavants. Our study is novel in the savant literature by clarifying the role of different traits and behaviours in the development of prodigious talent, in order to distinguish between previous theories that suggested the developmental pathway of the emergence of talent in autism. Our preliminary findings should be used to guide further research in delineating the direction and relative contribution of the factors identified in our study. Exploring further how these factors might influence different abilities (e.g. maths, music, art etc.) could be an important next step in our understanding of savant skills. Our current findings are important in defining savant syndrome as a legitimate sub-group of autism.

## Additional files

Additional file 1: **Table S1.** Details of Bayes factors calculated for the comparison of AQ scores between autistic-savants and autistic-nonsavants. (DOCX 22 kb)

Additional file 2: Calendar calculation strategy questionnaire. (DOCX 30 kb)

## Abbreviations

AQ: Autism Spectrum Quotient
CARD: Cambridge Autism Research Database
EPF: Enhanced perceptual functioning
LOI: Leyton Obsession Inventory
SCSQ: Sussex Cognitive Styles Questionnaire
SQ-R: Systemising Quotient-Revised
SSQ: Sussex Savant Questionnaire

## Footnotes

1 Calculation of a Bayes factor allows the evaluation of null results to determine whether the data supports evidence for the null against the alternative hypothesis [51]. Bayes factors are evaluated along a continuum although typically, a Bayes factor (BF) > .33 provides moderate support for the null hypothesis while a Bayes factor of > 3 provides moderate support for the alternative hypothesis, and values in between indicate no firm conclusions should be drawn.

2 We note that there were gender imbalances in our participant samples across groups (see [46] for example gender effects in autism). Given this, we repeated all analyses where we had found main effects (i.e. sensory sensitivity, obsessional traits, technical-spatial skills, and systemising) but this time ran ANCOVAs with gender entered as a covariate. Even after controlling for gender, all of our main effects were maintained: for sensory sensitivity (*F*(2, 111) = 28.06, *p* < .001, ηp2 = .34), obsessional traits (*F*(2, 111) = 7.74, *p* < .001, ηp2 = .13), technical-spatial skills (*F*(2, 111) = 6.36, *p* = .002, ηp2 = .11), and systemising (*F*(2, 91) = 22.09, *p* < .001, ηp2 = .34). In addition, the pattern of results for all our post hoc comparisons were maintained with autistic-savants scoring higher than both autistic-nonsavants and controls across all measures (all *p*’s < 0.05) while autistic-nonsavants scored higher than controls across all measures (*p*’s < .05) apart from obsessional traits and technical-spatial abilities (*p* > .05). In other words, gender had very little effect on our overall pattern of findings, and importantly, it had no effect whatsoever on our key findings comparing autistic-savants and autistic nonsavants.
