# Supplementary material for: Savant syndrome has a distinct psychological profile in autism
Source: Mol Autism. 2018 Oct 12;9:53. doi: 10.1186/s13229-018-0237-1 (PMC6186137; doi:10.1186/s13229-018-0237-1)
Supplement: Supplementary file 1 — Table S1. Details of Bayes factors calculated for the comparison of AQ scores between autistic-savants and autistic-nonsavants. (DOCX 22 kb) [file 13229_2018_237_MOESM1_ESM.docx]

**Additional File 1**

**Bayes factors for AQ data**

Table S1 shows the Bayes factors for each of the AQ domains (Social Skills, Attention Switching, Attention to Detail, Communication, Imagination) comparing our autistic-savants to our autistic-nonsavants. Bayes factors allow us to evaluate whether our null findings indicate lack of power or true support for the null hypothesis (Dienes, 2014). We calculated Bayes factors using an informative model of H1 (prior) assuming a half-normal distribution which was a study showing the difference between autistic individuals and winners of a UK Mathematics Olympiad using the same dependent measures of our own study (Baron-Cohen, Wheelwright, Skinner, Martin, & Clubley, 2001). Our Bayes analyses were run with the *Aladins Bayes fa*ctor *in R* package. This package requires: the half-normal parameter, the mean group difference from the comparable study, its degrees of freedom, the mean group difference in our current study, its standard error, and degrees of freedom. For example, these values for the Social Skills domain were, respectively, 0, 2.4, 72, -0.06970, .36112, 61. The outcome of our calculation of Bayes factors is shown in Row 3 of Table S1 below.

We next tested the sensitivity of our Bayes factors. This requires calculating an additional Bayes factor for our data modelled with an alternative H1 (prior). Here we adopt the recommended methods (see Dienes, 2014) in generating our H1 (again with a half-normal distribution) by taking into account not only the existing prior data (Baron-Cohen, Wheelwright, Skinner, Martin, & Clubley, 2001) but replacing our expected effect size (i.e., our “mean group difference from the comparable study” above) with an alternative value. For this we consider that the AQ has a maximum score of 10, which sets the boundary of the potential difference between savants and autistic participants. Thus, for the Social Skills factor for example, autistic participants scored 7.5 in our prior (Baron-Cohen et al., 2001) with the maximum score being 10 so our expected effect size for this factor is a maximum of 2.5. The protocol then requires we divide this value by 2 in order to produce a final plausible expected effect size for savants compared to autistic participants (i.e., 2.5/2 = 1.25). We did likewise for all factors and entered our parameters into Aladins in the same way as above (e.g., for Social Skills our full set of values were: 0, 1.25, -0.06970, .36112, 61). These resultant additional Bayes factors are shown in Row 4 of Table S1. A comparison of Rows 3 and 4 show that our two sets of Bayes factors give consistent results, both favouring H0 over H1 (with the exception of attention to detail). This indicates an acceptable level of robustness given our choice of informed prior. In summary, we conclude that there is moderate support for the null hypothesis (no differences between autistic-savants and autistic non-savants) for the AQ factors of Social Skills, Attention Switching, Communication, and Imagination with inconclusive results for Attention to Detail.

**Table S1** Bayes factors calculated for each AQ factor (columns 2-6). Rows 1 and 2 refer to the specific AQ domain while rows 3 and 4 show our calculated Bayes factors using an informed model of H1 with a t-distribution (row 3) and an alternative model of H1 (row 4).

|  | **AQ domain** | | | | |
| --- | --- | --- | --- | --- | --- |
|  | Social Skills | Attention Switching | Attention to detail | Communication | Imagination |
| Informative H1 | BF = 0.13 | BF = 0.05 | BF = 0.96 | BF = 0.06 | BF = 0.35 |
| Alternative H1 | BF = 0.24 | BF = 0.16 | BF = 0.26 | BF = 0.17 | BF = 0.30 |

**References**

Baron-Cohen, S., Wheelwright, S., Skinner, R., Martin, J., & Clubley, E. (2001). The Autism-Spectrum Quotient (AQ): Evidence from Asperger Syndrome/High-Functioning Autism, Males and Females, Scientists and Mathematicians. *Journal of Autism and Developmental Disorders*, *31*(1), 5–17. http://doi.org/10.1023/A:1005653411471

Dienes, Z. (2014). Using Bayes to get the most out of non-significant results. *Frontiers in Psychology*, *5*, 781. http://doi.org/10.3389/fpsyg.2014.00781
