# Supplementary material for: Savant syndrome has a distinct psychological profile in autism
Source: Mol Autism. 2018 Oct 12;9:53. doi: 10.1186/s13229-018-0237-1 (PMC6186137; doi:10.1186/s13229-018-0237-1)
Supplement: Supplementary file 2 — Calendar calculation strategy questionnaire. (DOCX 30 kb) [file 13229_2018_237_MOESM2_ESM.docx]

**Additional file 2**

**Calendar calculation strategy questionnaire**

We would just like to ask you a few final questions about your experience of learning to calendar calculate so far. Please indicate the extent to which you agree or disagree with the following statements

1. When calculating days of the week I used a visual strategy of picturing a calendar in my head.

Strongly Disagree Disagree Neither agree nor Disagree Agree Strongly Agree

2. I used the timeline on the computer screen (Mon, Tues… Sun) to move forwards and backwards between dates.

Strongly Disagree Disagree Neither agree nor Disagree Agree Strongly Agree

3. I used mental arithmetic to calculate days of the week (e.g. when adding and subtracting days).

Strongly Disagree Disagree Neither agree nor Disagree Agree Strongly Agree

4. I used rote memorization to remember days of the week (e.g. memorizing anchor dates).

Strongly Disagree Disagree Neither agree nor Disagree Agree Strongly Agree

5. If you are paying attention to this question then click neither agree nor disagree.

Strongly Disagree Disagree Neither agree nor Disagree Agree Strongly Agree

6. If you used any other kinds of strategies please let us know in the comment box below.

7. Overall, I found learning to calendar calculate easy.

Strongly Disagree Disagree Neither agree nor Disagree Agree Strongly Agree

8. Overall, I found learning to calendar calculate enjoyable.

Strongly Disagree Disagree Neither agree nor Disagree Agree Strongly Agree

9. I am keen to use this skill in my everyday life.

Strongly Disagree Disagree Neither agree nor Disagree Agree Strongly Agree

10. What made you want to learn how to calendar calculate? (Optional)

Is there anything else you would like to tell us about your participation in this study so far? (Optional)

Supplementary results

Descriptive statistics for our measures from Experiment 1 broken down according to the presence (+) or absence (-) of particular savant skills (calendar calculation was omitted due to insufficient participant numbers). Individual cells are displayed in the following format: Mean (SD), N.

|  | **AQ**  **(social skills)** | **AQ**  **(attention switching)** | **AQ**  **(attention to detail)** | **AQ**  **(communication)** | **AQ**  **(imagination)** | **SQ** | **GSQ** |
| --- | --- | --- | --- | --- | --- | --- | --- |
| **CONTROLS** | 3.64 (3.20), 28 | 5.25 (2.49), 28 | 4.82 (2.71), 28 | 2.36 (1.93), 28 | 2.68 (1.68), 28 | 50.96 (20.38), 27 | 41.68 (16.18), 31 |
| **AUTISTIC-NONSAVANT** | 8.57 (1.30), 30 | 8.87 (1.22), 30 | 7.43 (1.77), 30 | 8.40 (1.16), 30 | 6.73 (1.80), 30 | 74.58 (20.37), 33 | 68.22 (20.42), 36 |
| **AUTISTIC-SAVANT** |  |  |  |  |  |  |  |
| + maths ability | 8.40 (2.07), 10 | 8.20 (2.35), 10 | 8.20 (1.14), 10 | 7.90 (3.03), 10 | 6.00 (3.13), 10 | 89.25 (16.39), 8 | 81.50 (24.95), 16 |
| - maths ability | 8.18 (1.79), 28 | 8.79 (1.81), 28 | 7.39 (2.15), 28 | 7.96 (2.15), 28 | 6.25 (2.43), 28 | 89.46 (22.05), 28 | 78.14 (25.92), 36 |
|  |  |  |  |  |  |  |  |
| + musical ability | 7.83 (1.83), 6 | 8.67 (.82), 6 | 7.17 (2.48), 6 | 8.00 (1.79), 6 | 5.67 (1.37), 6 | 84.43 (20.95), 7 | 81.45 (21.66), 11 |
| - musical ability | 8.31 (1.86), 32 | 8.63 (2.11), 32 | 7.69 (1.87), 32 | 7.94 (2.49), 32 | 6.28 (2.76), 32 | 90.62 (20.83), 29 | 78.56 (26.56), 41 |
|  |  |  |  |  |  |  |  |
| + absolute pitch | 8.29 (1.50), 7 | 9.00 (.82), 7 | 7.43 (2.23), 7 | 8.43 (1.40), 7 | 7.00 (2.38), 7 | 86.38 (19.76), 8 | 79.42 (17.87), 12 |
| - absolute pitch | 8.23 (1.93), 31 | 8.55 (2.13), 31 | 7.65 (1.92), 31 | 7.84 (2.54), 31 | 6.00 (2.63), 31 | 90.29 (21.23), 28 | 79.10 (27.49), 40 |
|  |  |  |  |  |  |  |  |
| + art ability | 8.27 (1.85), 11 | 8.82 (1.33), 11 | 7.27 (2.15), 11 | 8.00 (2.14), 11 | 6.00 (2.32), 11 | 92.90 (22.94), 10 | 87.81 (28.54), 16 |
| - art ability | 8.22 (1.87), 27 | 8.56 (2.17), 27 | 7.74 (1.89), 27 | 7.93 (2.50), 27 | 6.26 (2.73), 27 | 88.08 (20.10), 26 | 75.33 (23.32), 36 |
|  |  |  |  |  |  |  |  |
| + memory | 8.16 (1.83), 19 | 8.11 (2.42), 19 | 7.63 (2.17), 19 | 7.95 (2.86), 19 | 6.16 (2.89), 19 | 94.59 (22.46), 17 | 80.35 (31.43), 26 |
| - memory | 8.32 (1.89), 19 | 9.16 (1.17), 19 | 7.58 (1.77), 19 | 7.95 (1.84), 19 | 6.21 (2.32), 19 | 84.79 (18.36), 19 | 78.00 (18.12), 26 |
|  |  |  |  |  |  |  |  |
| + mechanical skills | 9.00 (1.55), 6 | 9.17 (.98), 6 | 6.67 (2.25), 6 | 8.50 (1.97), 6 | 7.17 (1.94), 6 | 95.80 (19.88), 5 | 81.25 (13.86), 8 |
| - mechanical skills | 8.09 (1.87), 32 | 8.53 (2.08), 32 | 7.78 (1.88), 32 | 7.84 (2.45), 32 | 6.00 (2.68), 32 | 88.39 (20.96), 31 | 78.80 (27.11), 44 |
|  |  |  |  |  |  |  |  |
| + language learning | 8.88 (1.73), 8 | 8.63 (1.30), 8 | 7.50 (1.93), 8 | 8.63 (2.00), 8 | 6.50 (1.41), 8 | 94.50 (24.23), 8 | 85.42 (27.05), 12 |
| - language learning | 8.07 (1.86), 30 | 8.63 (2.11), 30 | 7.63 (1.99), 30 | 7.77 (2.46), 30 | 6.10 (2.83), 30 | 87.96 (19.84), 28 | 77.30 (24.98), 40 |
|  | **SCSQ**  **(imagery ability)** | **SCSQ**  **(technical spatial)** | **SCSQ**  **(language)** | **SCSQ**  **(organisation)** | **SCSQ**  **(global bias)** | **SCSQ**  **(systemising)** |  |
| **CONTROLS** | 3.20 (.65), 31 | 2.85 (.72), 31 | 3.66 (.71), 31 | 2.94 (.88), 31 | 3.27 (.66), 31 | 2.74 (.55), 31 |  |
| **AUTISTIC-NONSAVANT** | 3.27 (.94), 36 | 2.94 (.65), 36 | 3.76 (.79), 36 | 3.59 (.67), 36 | 2.37 (.59), 36 | 3.11 (.57), 36 |  |
| **AUTISTIC-SAVANT** |  |  |  |  |  |  |  |
| + maths ability | 3.60 (.94), 16 | 3.89 (.62), 16 | 4.43 (.34), 16 | 3.58 (.57), 16 | 2.38 (.86), 16 | 3.54 (.54), 16 |  |
| - maths ability | 3.55 (.87), 36 | 3.37 (.80), 36 | 3.97 (.97), 36 | 3.57 (.83), 36 | 2.37 (.81), 36 | 3.24 (.58), 36 |  |
|  |  |  |  |  |  |  |  |
| + musical ability | 3.45 (.77), 11 | 3.34 (.76), 11 | 4.00 (.98), 11 | 3.48 (.65), 11 | 2.56 (.67), 11 | 3.14 (.43), 11 |  |
| - musical ability | 3.59 (.92), 41 | 3.59 (.79), 41 | 4.14 (.82), 41 | 3.60 (.79), 41 | 2.32 (.85), 41 | 3.39 (.60), 41 |  |
|  |  |  |  |  |  |  |  |
| + absolute pitch | 3.29 (.97), 12 | 3.41 (.82), 12 | 4.04 (.86), 12 | 3.32 (.70), 12 | 2.61 (.80), 12 | 3.36 (.41), 12 |  |
| - absolute pitch | 3.64 (.85), 40 | 3.57 (.78), 40 | 4.13 (.86), 40 | 3.65 (.76), 40 | 2.30 (.82), 40 | 3.33 (.62), 40 |  |
|  |  |  |  |  |  |  |  |
| + art ability | 4.04 (.58), 16 | 3.75 (.82), 16 | 4.24 (.83), 16 | 3.61 (.61), 16 | 2.16 (.62), 16 | 3.28 (.58), 16 |  |
| - art ability | 3.35 (.92), 36 | 3.44 (.76), 36 | 4.05 (.87), 36 | 3.56 (.82), 36 | 2.47 (.88), 36 | 3.36 (.58), 36 |  |
|  |  |  |  |  |  |  |  |
| + memory | 3.62 (.86), 26 | 3.66 (.88), 26 | 4.20 (.81), 26 | 3.58 (.71), 26 | 2.32 (.82), 26 | 3.49 (.59), 26 |  |
| - memory | 3.50 (.92), 26 | 3.41 (.67), 26 | 4.02 (.89), 26 | 3.58 (.81), 26 | 2.42 (.83), 26 | 3.18 (.53), 26 |  |
|  |  |  |  |  |  |  |  |
| + mechanical skills | 4.00 (.63), 8 | 4.08 (.49), 8 | 3.79 (.85), 8 | 3.54 (.81), 8 | 2.19 (.62), 8 | 3.27 (.42), 8 |  |
| - mechanical skills | 3.48 (.90), 44 | 3.43 (.79), 44 | 4.17 (.85), 44 | 3.58 (.76), 44 | 2.40 (.85), 44 | 3.34 (.60), 44 |  |
|  |  |  |  |  |  |  |  |
| + language learning | 3.67 (.82), 12 | 3.83 (.67), 12 | 4.43 (.62), 12 | 3.69 (.78), 12 | 2.57 (.72), 12 | 3.22 (.72), 12 |  |
| - language learning | 3.53 (.91), 40 | 3.44 (.80), 40 | 4.01 (.89), 40 | 3.54 (.76), 40 | 2.31 (.84), 40 | 3.37 (.53), 40 |  |

|  | **LOI**  **(contamination)** | **LOI (doubts/repeating)** | **LOI**  **(checking/detail)** | **LOI**  **(worries/just right)** |
| --- | --- | --- | --- | --- |
| **CONTROLS** | 1.00 (1.46), 31 | 1.68 (2.01), 31 | 2.55 (2.05), 31 | .52 (.63), 31 |
| **AUTISTIC-NONSAVANT** | 2.36 (1.61), 36 | 3.44 (2.38), 36 | 4.14 (1.62), 36 | .75 (.55), 36 |
| **AUTISTIC-SAVANT** |  |  |  |  |
| + maths ability | 2.69 (2.82), 16 | 3.13 (2.33), 16 | 4.25 (1.81), 16 | .94 (.85), 16 |
| - maths ability | 3.00 (2.33), 36 | 3.22 (2.09), 36 | 4.50 (1.66), 36 | 1.19 (.92), 36 |
|  |  |  |  |  |
| + musical ability | 2.18 (1.78), 11 | 4.27 (2.00), 11 | 4.36 (1.69), 11 | .82 (.75), 11 |
| - musical ability | 3.10 (2.61), 41 | 2.90 (2.11), 41 | 4.44 (1.72), 41 | 1.20 (.93), 41 |
|  |  |  |  |  |
| + absolute pitch | 1.42 (1.16), 12 | 4.08 (1.83), 12 | 4.08 (1.31), 12 | 1.00 (.85), 12 |
| - absolute pitch | 3.35 (2.59), 40 | 2.93 (2.18), 40 | 4.53 (1.80), 40 | 1.15 (.92), 40 |
|  |  |  |  |  |
| + art ability | 3.19 (2.51), 16 | 2.63 (2.19), 16 | 4.44 (1.46), 16 | 1.19 (.98), 16 |
| - art ability | 2.78 (2.47), 36 | 3.44 (2.10), 36 | 4.42 (1.81), 36 | 1.08 (.87), 36 |
|  |  |  |  |  |
| + memory | 3.00 (2.68), 26 | 3.23 (2.25), 26 | 4.35 (1.85), 26 | 1.12 (.86), 26 |
| - memory | 2.81 (2.28), 26 | 3.15 (2.07), 26 | 4.50 (1.56), 26 | 1.12 (.95), 26 |
|  |  |  |  |  |
| + mechanical skills | 2.75 (3.37), 8 | 2.88 (2.80), 8 | 4.88 (1.81), 8 | 1.25 (.71), 8 |
| - mechanical skills | 2.93 (2.32), 44 | 3.25 (2.04), 44 | 4.34 (1.68), 44 | 1.09 (.94), 44 |
|  |  |  |  |  |
| + language learning | 4.08 (3.00), 12 | 3.92 (2.39), 12 | 5.25 (1.60), 12 | 1.25 (1.14), 12 |
| - language learning | 2.55 (2.21), 40 | 2.98 (2.04), 40 | 4.18 (1.66), 40 | 1.08 (.83), 40 |
